# An assessment of potential biomarkers of environment enteropathy and its association with age and microbial infections among children in Bangladesh

**DOI:** 10.1371/journal.pone.0250446

**Published:** 2021-04-22

**Authors:** Muhammad Ikhtear Uddin, Motaher Hossain, Shahidul Islam, Aklima Akter, Naoshin Sharmin Nishat, Tasnin Akter Nila, Tanzeem Ahmed Rafique, Daniel T. Leung, Stephen B. Calderwood, Edward T. Ryan, Jason B. Harris, Regina C. LaRocque, Taufiqur Rahman Bhuiyan, Firdausi Qadri

**Affiliations:** 1 Infectious Diseases Division, icddr,b, Dhaka, Bangladesh; 2 Division of Infectious Diseases, University of Utah School of Medicine, Salt Lake City, Utah, United States of America; 3 Division of Infectious Diseases, Massachusetts General Hospital, Boston, Massachusetts, United States of America; 4 Department of Medicine, Harvard Medical School, Boston, Massachusetts, United States of America; 5 Department of Microbiology, Harvard Medical School, Boston, Massachusetts, United States of America; 6 Department of Immunology and Infectious Diseases, Harvard T.H. Chan School of Public Health, Boston, Massachusetts, United States of America; 7 Department of Pediatrics, Harvard Medical School, Boston, Massachusetts, United States of America; Universiti Putra Malaysia, MALAYSIA

## Abstract

Interventional studies targeting environment enteropathy (EE) are impeded by the lack of appropriate, validated, non-invasive biomarkers of EE. Thus, we aimed to validate the association of potential biomarkers for EE with enteric infections and nutritional status in a longitudinal birth cohort study. We measured endotoxin core antibody (EndoCab) and soluble CD14 (sCD14) in serum, and myeloperoxidase (MPO) in feces using commercially available enzyme-linked immunosorbent assay (ELISA) kits. We found that levels of serum EndoCab and sCD14 increase with the cumulative incidence of enteric infections. We observed a significant correlation between the fecal MPO level in the children at 24 months of age with the total number of bacterial and viral infections, the total number of parasitic infections, and the total number of diarrheal episodes and diarrheal duration. We observed that the levels of serum EndoCab, sCD14, and fecal MPO at 3 months of age were significantly associated with whether children were malnourished at 18 months of age or not. Biomarkers such as fecal MPO, serum EndoCab and sCD14 in children at an early age may be useful as a measure of cumulative burden of preceding enteric infections, which are predictive of subsequent malnutrition status and may be useful non-invasive biomarkers for EE.

## Introduction

Inadequate sanitation and hygiene levels are commonly observed among people living in low and middle-income countries (LMICs) [1–7]. Such compromised environmental conditions predispose infants and children to recurrent exposure to multiple enteric pathogens, leading to environmental enteropathy (EE). EE is a subclinical condition characterized by blunting of intestinal villi, impaired gut immune function, and increased intestinal permeability and is hypothesized to be responsible for subsequent malnutrition, stunting of growth, reduced oral vaccine efficacy, and possibly lower protective immunity following natural diseases in children [4, 8–11]. According to the recent national survey in Bangladesh among children under five years of age, 36% of children were stunted, 33% were underweight, and 14% were wasted based on WHO standards [12]. Stunting affects at least 150.8 million children, and wasting affects at least 50.5 million children under five years old globally [13]. Oral vaccines for polio, rotavirus, typhoid, and cholera are less immunogenic or less protective among children living in LMICs compared to high-income countries (HICs), which consequently compromises their potential as effective public health interventions; EE is suggested to be one of the causes for this phenomenon [2, 11, 14–17].

Studies of EE have been impaired by a lack of validated non-invasive biomarkers, and hence judging the efficacy of interventions to prevent or treat EE is hindered [18]. As biopsies are invasive, investigators are trying to identify indirect and non-invasive biomarkers of EE [19]. EE has been frequently assessed by dual sugar absorption/permeability tests (Lactulose to Mannitol ratio) to assess the permeability of the gut to macro-molecules and intestinal absorptive capacity. Although this test is non-invasive and clinically significant, it is costly and takes significant time to perform. Moreover, the results obtained from different studies were not comparable [20–22]. Thus, assessment of potential serum and fecal biomarkers may provide an alternative, easier approaches to evaluate and follow EE.

Endotoxin core antibody (EndoCab), a marker of microbial translocation, measures antibodies against lipopolysaccharide (LPS) and reflects a loss of epithelial cell surface integrity and hyperstimulation by intestinal bacteria [2, 19, 23, 24]. Another potential biomarker of EE is soluble CD14 (sCD14), a glycoprotein primarily produced by macrophages, which stimulates pro-inflammatory responses against gram-negative bacteria. sCD14 was previously assessed as a marker of systemic inflammation [11, 25, 26]. Myeloperoxidase (MPO) has been extensively used as a marker of intestinal inflammation because of less influences by variable factors such as breast milk and age [24–27].

EndoCab [19, 21], sCD14 [28], and MPO [6] have been previously postulated to be biomarkers of EE. In this study, we aimed to assess these previously identified biomarkers of EE and study their association with enteric infections and malnutrition in young children followed longitudinally from birth in Bangladesh to validate their use in future studies.

## Materials and methods

### Study participants and ethics statement

The serum and fecal samples used in this study were taken from a previously described birth cohort study conducted in 2002–2004 in an urban slum area of Mirpur, Dhaka, Bangladesh [29]. In this first study, 695 pregnant mothers were screened, and 321 children were enrolled considering the inclusion criteria (i.e. normal delivery with no congenital abnormalities and with a birth weight of ≥ 2 kg). The assays for our current study were conducted in 2013–2014. The subsamples that were used in this study were selected in an unbiased manner. Out of 321 participants in our previous birth cohort study, we selected all 43 individuals based on the availability of sufficient serum and fecal material from all three-time points (3 months, 12 months and 24 months) to complete the laboratory measurements of serum EndoCab and sCD14, and fecal MPO. The sample size is variable in analyses because all of these 43 individuals did not include all kinds of assays. Infants were enrolled at birth and were followed until two years of age in the previous study [29]. Written informed consent was obtained from the parents or legal guardians of the participants [29]. The institutional review board of the International Centre for Diarrhoeal Disease Research, Bangladesh (icddr,b) approved the study.

### Demographic details

Field Research Assistants (FRAs) visited each child every other day and collected information on diarrhea and other illnesses using a structured questionnaire. The child was referred to the study clinic if the FRA found the child was experiencing an acute illness. Parents or guardians of the study participants were encouraged to visit the study clinic for medical assistance if the study child became sick. Anthropometric data were taken at the time of enrollment and 3-months intervals. The weight and length of infants were measured using standard techniques described previously [9]. Anthropometric data analyses are described in our previous publication [29].

### Specimen collection, transport, and pathogen detection

Blood and fecal specimens were taken from the enrolled children at three different time points (3, 12, and 24 months of age) and during diarrheal episodes. Diarrhea was operationally defined as ≥ 3 unformed stools within a 24-hours period, as described in our previous study [29]. Two episodes of diarrhea were considered different when they were separated by at least 3 diarrhea-free days. We collected venous blood from each participant in a vacutainer blood collection tube (Becton Dickinson), processed for separation of serum, and stored in aliquots at -20°C. Stool specimens and rectal swabs, were collected in Cary Blair medium and transported to the icddr,b laboratory within 4 to 5 hours of collection. Collected stool samples were screened for common enteric bacterial pathogens, including *Vibrio cholerae* O1/O139, *Campylobacter jejuni*, *Salmonella* spp., and *Shigella* spp. by following standard laboratory techniques [29–31]. Rotavirus was tested by ELISA [29, 32], and enteric parasites (Giardia, Ascaris, and Trichuris) were detected by microscopic examination of stools [29]. Then we counted the number of independent bacterial and viral infections manually.

### Anthropometric analyses

We assessed the nutritional status of the children at 3-months intervals. The children were defined as malnourished when they were underweight (weight for age Z [WAZ] score < -2) or stunted growth (height for age Z [HAZ] score < -2) or wasted (weight for height Z [WHZ] score < -2) and as nourished when the WAZ/HAZ/WHZ score was ≥-2 based on WHO standard [29].

### Laboratory procedures

Commercially available ELISA kits were used to assay endotoxin core antibody (EndoCab Human IgG; Hycult Biotech, Uden, Netherlands) and soluble CD14 (sCD14; Quantikine ELISA) in serum specimens, and myeloperoxidase (MPO) in stool specimens (Immundiagnostik, Bensheim, Germany) at three different time points. All these assays were carried out according to the manufacturers’ descriptions with some modifications, as stated previously [25]. Brifely, Plasma samples were diluted at a ratio of 1:1000 and 1:200 for measuring sCD14 and EndoCAb. The 1:200 was used to measure the MPO level in fecal extracts. We had run our samples with standards in a duplicate manner. If any standard values outside of the manufacturers’ descriptions were found, we repeated the corresponding samples or excluded those from our analysis. For ELISA procedures, Eon microplate reader was used (BioTek, USA), to measure absorbances, and Gen5 software was used to calculate concentrations of markers unless stated otherwise.

### Statistical analyses

We performed statistical analyses using Statistical Package for Social Sciences (SPSS) version 20.0 (SPSS Inc., Chicago, IL) and GraphPad Prism 5.0 (GraphPad Software, Inc., La Jolla, CA). Correlations were tested by Spearman correlation test available in SPSS package, and Kruskal-Wallis and Mann Whitney U tests were done for comparison between time points using GraphPad Prism (GraphPad Software, Inc., La Jolla, CA).

## Results

### Demographics of study participants

Demographics of the study participants, along with the socioeconomic status and breastfeeding length, are described in Table 1.

**Table 1:** Demographic characteristics of the study participants.

Features		Results of Features
Total no. of children		43
Gender	No. (%) of Male	24 (55.81)
	No. (%) of Female	19 (44.17)
Median weight at birth		2.70 gm
Median height at birth		48.50 cm
No. (%) of malnourished children at birth (WHZ <-2)		2 (5.40)
No. (%) of malnourished children at 24 months (WHZ <-2)		9 (24.30)
No. of children (%) with ≥ 6 months of breastfeeding		3 (6.98)
No. of children (%) with ≤ 6 months of breastfeeding		40 (93)
**Socioeconomic Status**		
Average no. of family members/household		5
No. (%) of families with monthly income ≤ $60 US		33 (76.7)
Housing	Thrash (%)	11 (25.6)
	Tin shaded (%)	23 (53.5)
	Building (%)	9 (20.9)
Drinking water	Boiled (%)	11 (25.6)
	Treated (%)	0 (0)
	Not boiled (%)	32 (74.4)
Latrines	Sanitary but Public (%)	22 (51.2)
	Sanitary but Personal (%)	12 (27.9)
	Others (%)	9 (20.9)

### Levels of serum EndoCab and sCD14 increased with age over a two-year period

**Fig 1:**
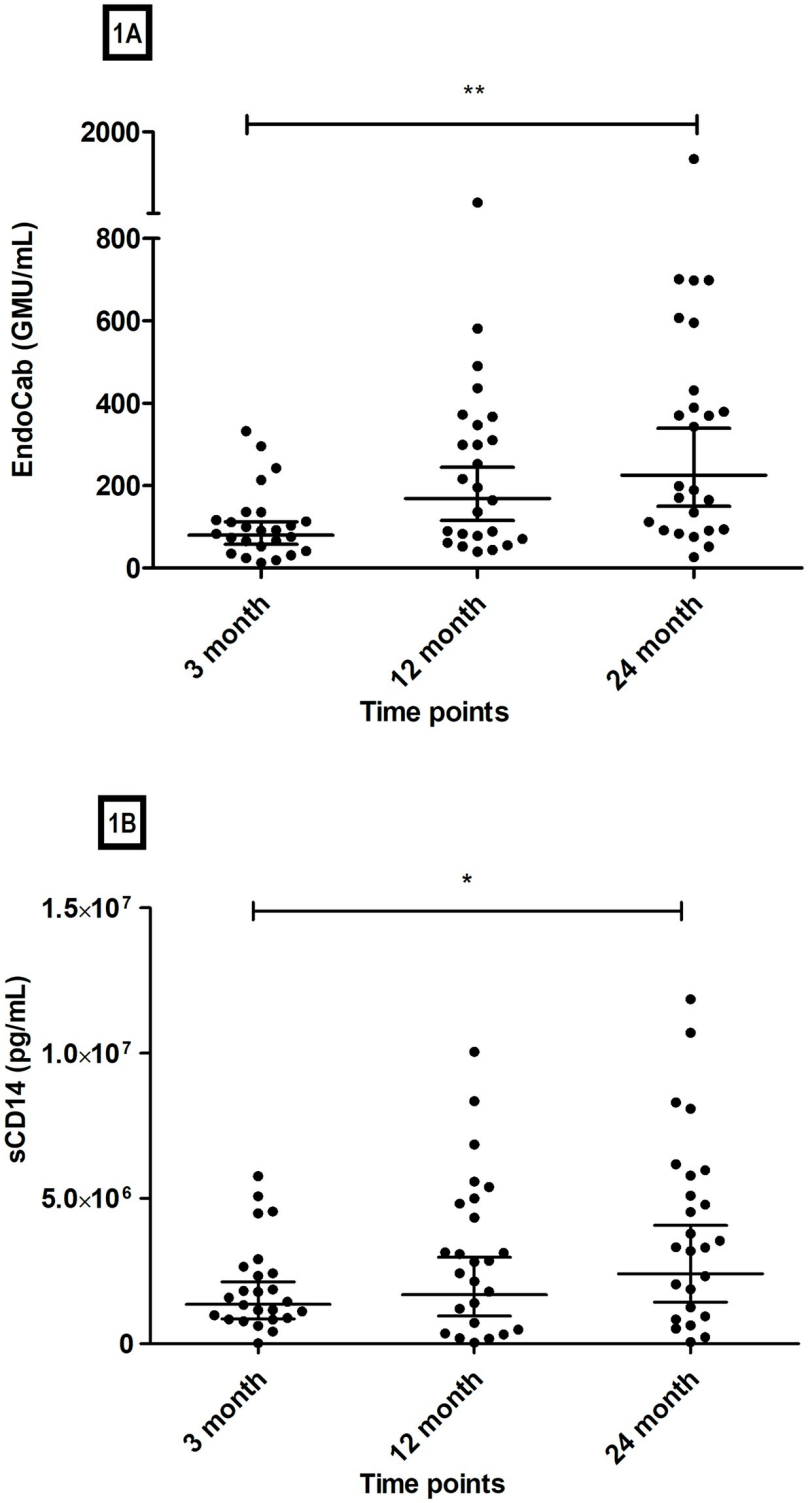
Levels of EndoCab and sCD14 at different time points (n = 25). Y-axis indicates EndoCab levels (1A) and sCD14 levels (1B), while X-axes indicate time points after birth (1A and 1B). Each single dot represents an individual EndoCab level (GMU/mL) (1A) and sCD14 level (pg/mL) (1B) at that time point. The horizontal bars denote the geometric mean values, and the error bars represent the 95% confidence intervals (CI). We performed the Kruskal-Wallis test for statistical evaluation [**P = 0.001(1A) and *P < 0.05 (1B)].

We measured IgG EndoCab and sCD14 levels in serum specimens among 25 children at three different time points (3, 12, and 24 months) (Fig 1). Levels of EndoCab (GMU/mL) increased with age (Fig 1A) and were significantly higher at 24 months compared to 3 months (*P* = 0.001). Levels of sCD14 (pg/mL) also increased with age (Fig 1B) and were significantly higher at 24 months compared to 3 months (*P* < 0.05).

### Correlation of total number of bacterial and viral infections with the level of fecal MPO at 24 months of age

**Fig 2:**
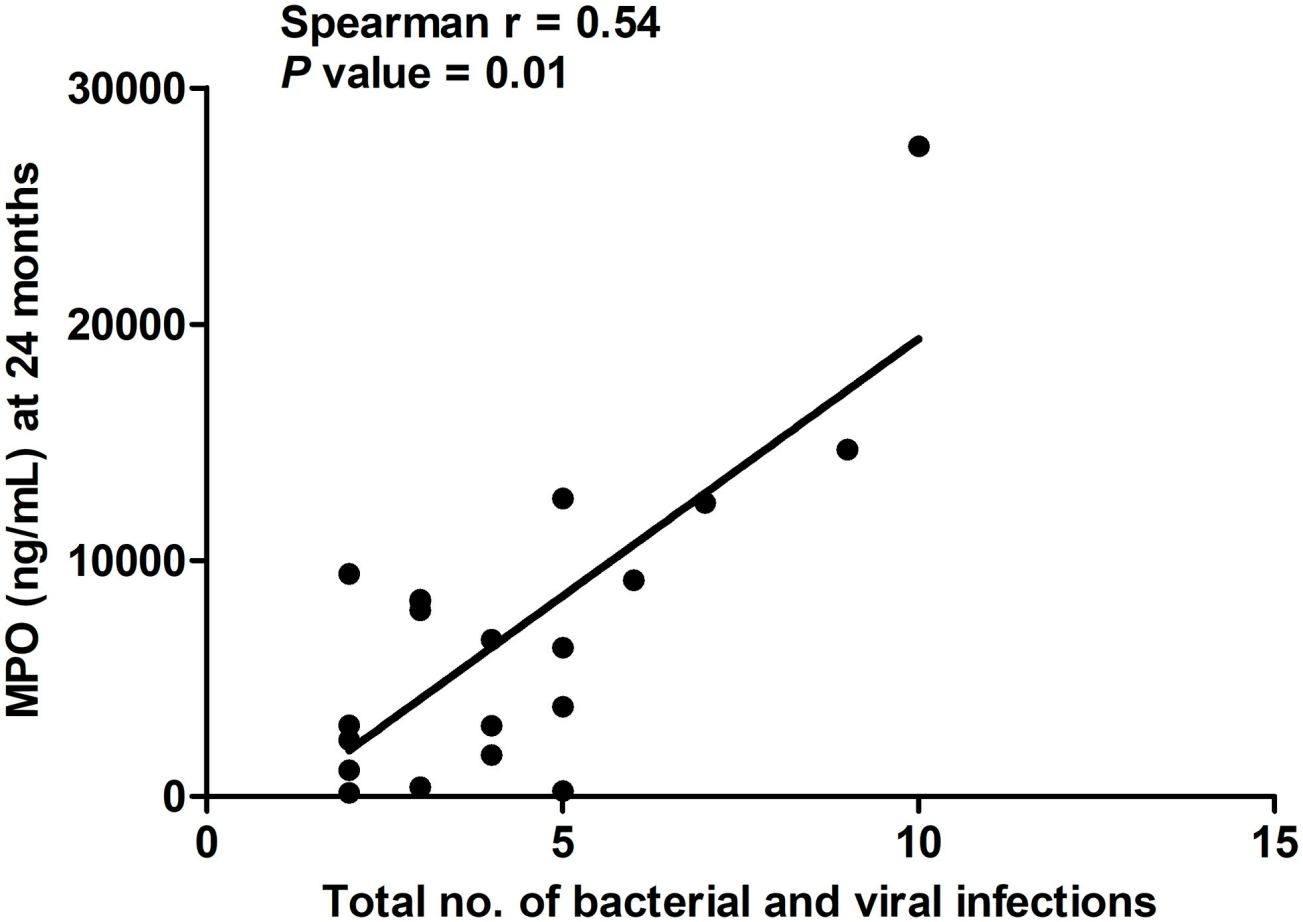
Correlation of the total number of bacterial and viral infections (n = 20) with fecal MPO levels at 24 months. The X-axis indicates the total number of bacterial and viral infections, while the Y-axis denotes fecal MPO levels (ng/mL) at 24 months of age. We performed the Spearman correlation test for statistical evaluation (r = 0.54 and *P* = 0.01).

We quantified MPO levels in fecal specimens and determined the correlation of fecal MPO levels at 24 months of age with the total number of bacterial and viral infections in each child over that period (Fig 2). We observed a moderate correlation between the total number of bacterial and viral infections with the MPO level in the children at 24 months of age (r = 0.54, *P* = 0.01); however, we did not find any significant correlation with the children who had less than 5 infections (r = 0.11, *P* = 0.72). The median (range) of the total number of bacterial and viral infections was 4 (2–10) in the 20 participants. We also analyzed the correlation of fecal MPO levels at 3 and 12 months of age, with the total number of bacterial and viral infections in each child but did not find any significant correlation. We analyzed the correlation of the total number of bacterial and viral infections with the level of IgG EndoCab and sCD14 in serum at 24 months but did not find any significant correlation.

### Correlation between the total number of parasitic infections and levels of MPO at 24 months of age

**Fig 3:**
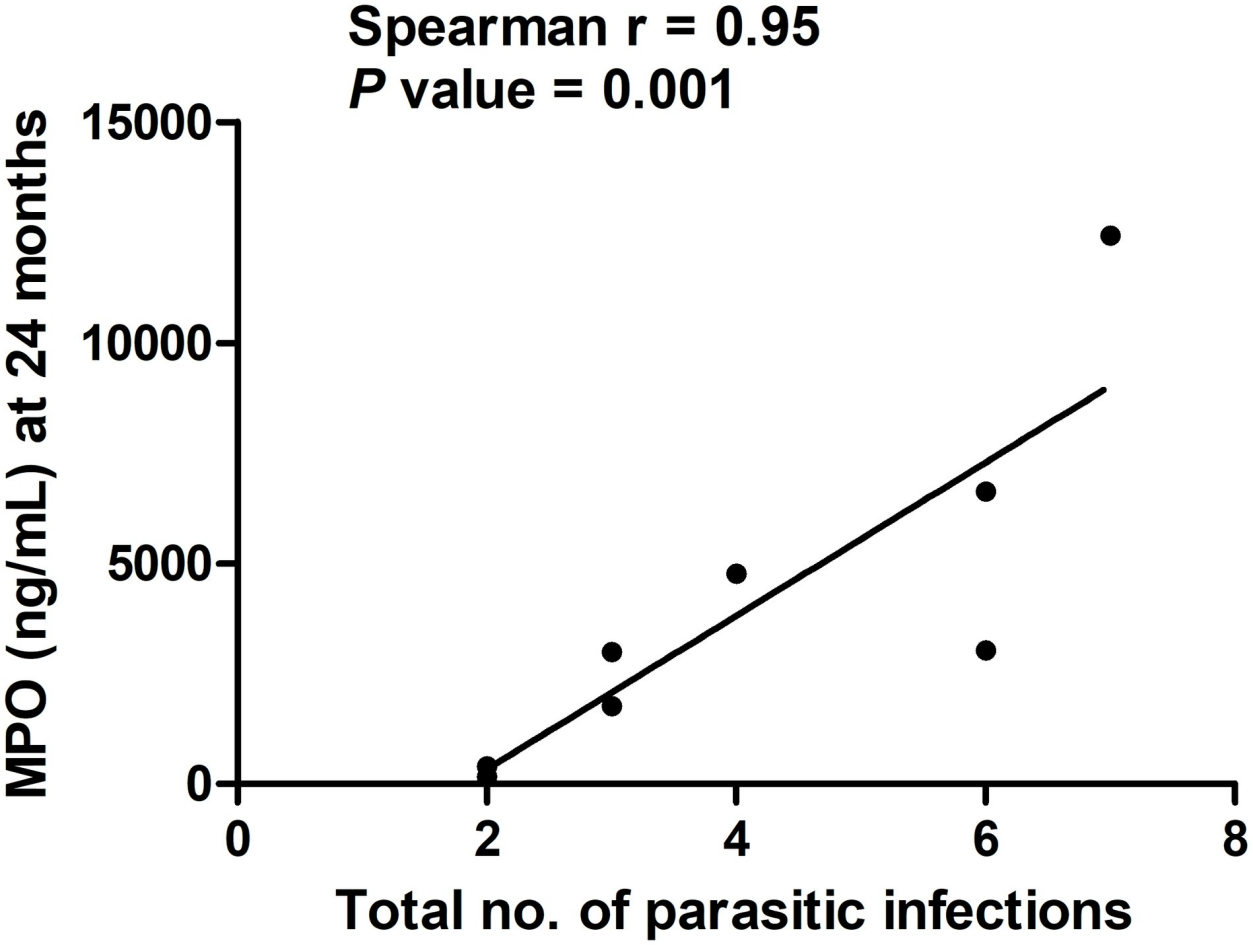
Correlation of the total number of parasitic infections (n = 8) with fecal MPO levels at 24 months. The X-axis indicates the total number of parasitic infections, while the Y-axis denotes fecal MPO levels (ng/mL) at 24 months of age. We performed the Spearman correlation test for statistical evaluation (r = 0.95 and *P* = 0.001).

We analyzed fecal MPO levels to study their association with parasitic infections (Fig 3). We observed a strong correlation between the total number of parasitic infections in each child and MPO levels (ng/mL) at 24 months in stool specimens from that child (r = 0.95, *P* = 0.001), however, we did not notice any correlation at 3 and 12 months. The median (range) of the total number of parasitic infections was 3.5 (2–7) in 8 participants. We also analyzed the correlation of the total number of parasitic infections with levels of IgG EndoCab and sCD14 in serum at 24 months but did not find any significant correlation.

### Correlation of diarrheal duration and the total number of diarrheal episodes with fecal MPO levels at 24 months of age

**Fig 4:**
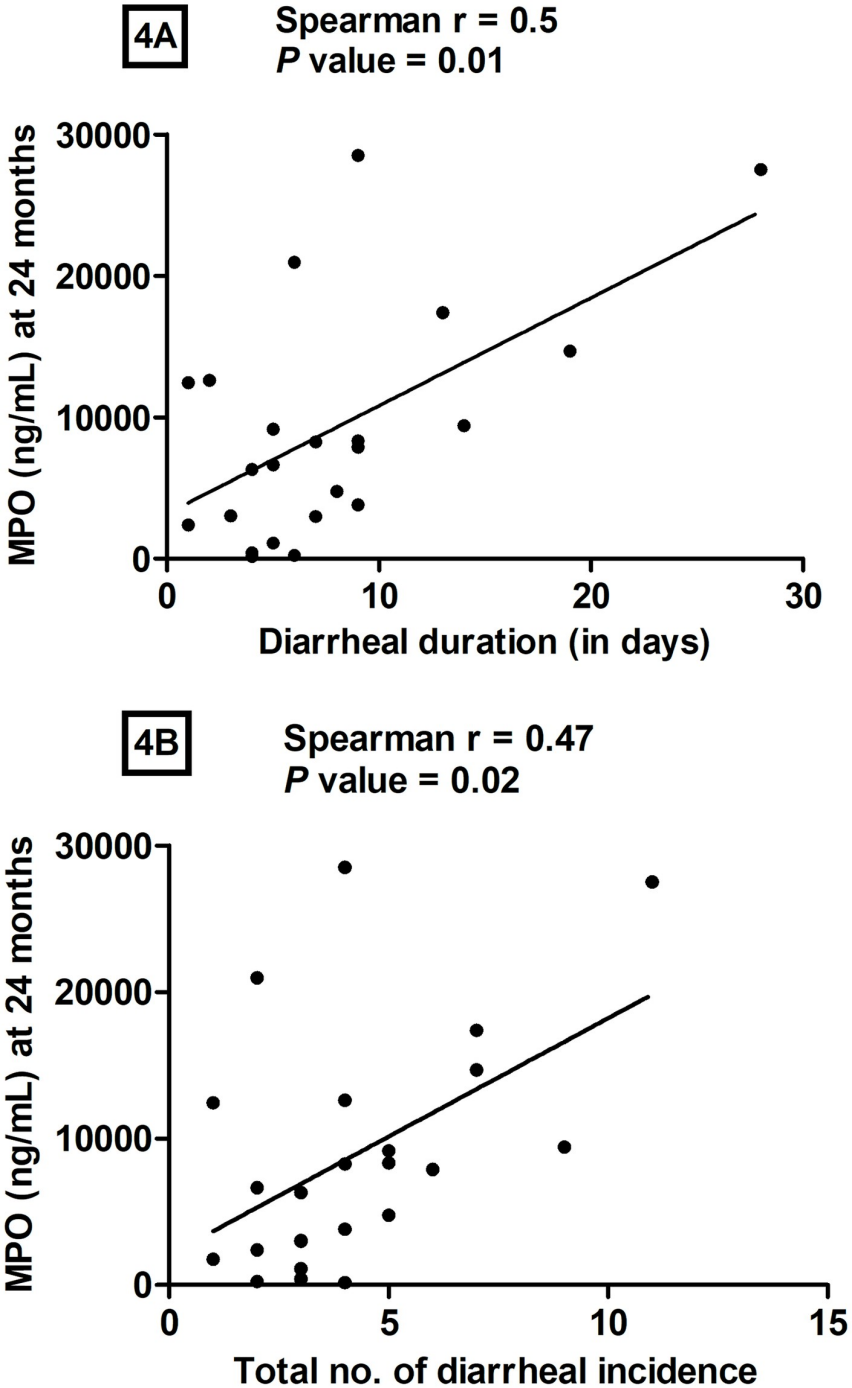
Correlation of diarrheal duration (n = 23) and the total number of diarrheal episodes (n = 24) with fecal MPO levels at 24 months. The X-axis indicates diarrheal duration (4A) and the number of individual diarrheal episodes (4B), while the Y-axis denotes MPO levels (ng/mL) at 24 months of age (4A and 4B). We performed the Spearman correlation test for statistical evaluation [r = 0.5 and *P* = 0.01(4A), r = 0.47 and *P* = 0.02(4B)]. Diarrheal duration denotes the total number of days over the two years when a child had diarrhea cumulatively.

We analyzed fecal MPO levels at 24 months to investigate whether there was an association of MPO levels with the total number of days of diarrhea or the total number of individual diarrheal episodes in each child (Fig 4). We found a significant correlation of MPO levels (ng/mL) in fecal specimens at 24 months with both total diarrheal duration (r = 0.5, *P* = 0.01) (Fig 4A), and the total number of diarrheal episodes in a child (r = 0.47, *P* = 0.02) (Fig 4B), but did not notice any correlation at 3 and 12 months. We also analyzed the correlation of MPO levels (ng/mL) in fecal specimens at 24 months with the children who had a diarrheal duration of less than 10 days; however, we did not observe any significant correlation (r = 0.20, *P* = 0.40). The median (range) of the total number of days of diarrhea in 23 participants was 6 days (1–28 days). The median (range) of the total number of individual diarrheal episodes in each child was 4 (1–11) in 24 participants. We also analyzed the correlation of the total number of days of diarrhea or the total number of individual diarrheal episodes in each child with levels of IgG EndoCab and sCD14 in serum but did not find any significant correlation.

### Association of serum EndoCab, sCD14, and fecal MPO levels with nutritional status

**Fig 5:**
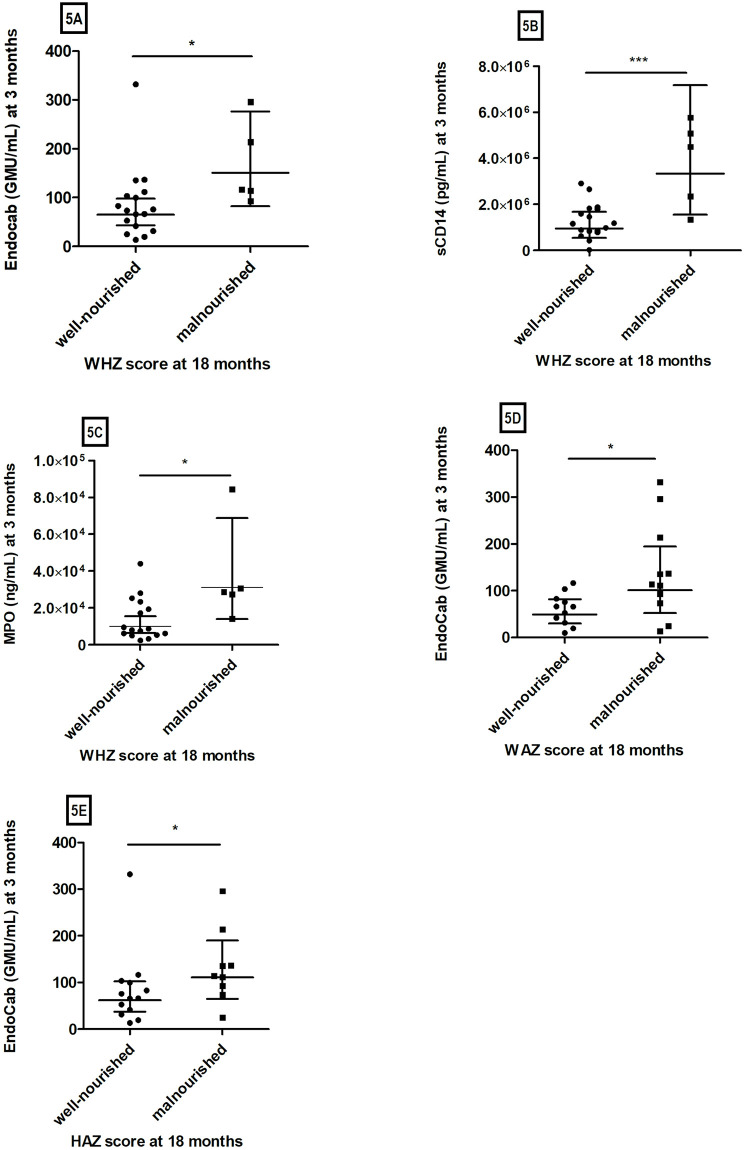
Association of serum EndoCab, sCD14, and fecal MPO levels with nutritional status. The X-axes show nutritional status (5A, 5B, 5C, 5D and 5E) and the Y-axes show EndoCab concentrations (GMU/mL) (5A, 5D and 5E), sCD14 levels(5B), and MPO levels (5C) at 3 months. Data are presented as geometric mean with 95% confidence intervals (CI). We performed the Mann Whitney test for statistical analysis of the data [*P = 0.03(5A), ***P = 0.001(5B), *P = 0.01(5C), *P = 0.03(5D), and *P = 0.05(5E)].

We analyzed the concentrations of EndoCab and sCD14 in serum, as well as MPO levels in fecal specimens of children at three months of age, to see if the level of serum and fecal markers at 3 months of age predicts nutritional status at 18 months of age (Fig 5). We found that the EndoCab concentration (GMU/mL) at 3 months of age was significantly higher in the children who were wasted (malnourished) at the age of 18 months than in those who were well-nourished at that time point (*P* = 0.03) (Fig 5A). We found similar results for serum sCD14 levels (pg/mL) (*P* = 0.001) (Fig 5B) and fecal MPO levels (ng/mL) (*P* = 0.01) at 3 months and subsequent malnutrition (wasting) (Fig 5C).

We also found that the levels of EndoCab at 3 months of age were considerably elevated in children who were subsequently underweight or stunted (malnourished) compared to the children who were not underweight or stunted (well-nourished) at 18 months of age (*P* = 0.03, Fig 5D; *P* = 0.05, Fig 5E).

## Discussion

The serum antibody against endotoxin is known as the endotoxin core antibody (EndoCab). Endotoxin is a high molecular weight complex of LPS that is a part of the gram-negative bacterial outer membrane [2]. EndoCab is produced by B cells in a T-cell independent manner after LPS exposure [2]. It also plays an essential role in acute inflammation. The levels of EndoCab are more important than the direct measurement of endotoxin levels in determining exposure to endotoxin [33]. Elevated plasma EndoCab IgG titers reflect exposure to endotoxin that has translocated across a leaky gut in response to repeated bacterial enteric infections [19, 23]. In addition to bacterial infection, EndoCab levels in serum are increased following certain parasitic infections, including *Strongyloides stercoralis* and hookworm infection, presumably because of gut translocation from intestinal bacteria [34, 35]. Higher EndoCab titers were previously observed among children living in contaminated households compared to children living in a clean household environment [19]. Elevated serum EndoCab has also been associated with Gambian infants with impaired growth [2, 4, 21, 26]. In our study, we used longitudinal follow-up of a birth cohort to define this relationship better. We found that levels of EndoCab increased over time in children and were significantly higher at 24 months compared to 3 months of age, which is consistent with other study findings [21].

sCD14 is a glycoprotein and an essential component of the innate immune system. CD14 is expressed on the cell surface of mainly monocytes and macrophages and, to a lesser extent, by neutrophils and dendritic cells [36]. It acts as a co-receptor, along with Toll-like receptor 4 (TLR-4) and myeloid differentiation factor 2 (MD-2), for the detection of endotoxin (LPS) from gram-negative bacteria in the presence of lipopolysaccharide-binding protein (LBP) [37–40]. In cells that do not express cell surface CD14, sCD14 can also play the essential role in immune response of cells to LPS, and the level of sCD14 increases during infection and inflammation [41, 42]. In this study, we found that the levels of sCD14 increased over the study period among study children. This increase might be associated with recurrent exposure to enteric infections. Several studies have evaluated sCD14 as a marker of LPS translocation that results from enteric infection and its ability to predict subsequent growth impairment [11, 25, 26]. Our previous work also showed that the level of sCD14 is higher in 6 to 14 years old Bangladeshi children than 3–5 years old children [25].

MPO is an innate immunity enzyme released from neutrophils, monocytes and macrophages at low concentration [43]. MPO is excreted in feces, and it is stable in fecal matter, where its measurement can provide evidence of intestinal inflammation [44, 45]. A good correlation has been observed between fecal MPO levels and other gut inflammation markers in previous studies. For example, in an active inflammatory bowel disease, fecal MPO levels increase significantly and correlate with disease activity [43]. Another group recently demonstrated a correlation between fecal MPO levels and the Lactulose to Mannitol ratio [26] and postulated MPO as a promising biomarker of EE. In this study, we found a significant correlation of MPO levels at 24 months of age with the total number of bacterial and viral infections in each child. Our finding is consistent with the prior findings that showed that MPO levels increase along with the number of pathogens found in an individual [26, 46]. This would be expected because frequent infections disrupt intestinal barrier function, which allows translocation of macromolecules across the mucosa and eventually causes mucosal inflammation [10, 21, 47]. Our findings of children followed serially over time provide direct evidence of an association between enteric infections and elevated fecal MPO, which is consistent with other study findings [6].

Parasitic infections with *Giardia intestinalis*, *Ascaris lumbricoides*, *Trichuris trichiura*, and other geohelminths are widespread in many tropical environments [9, 10, 48, 49]. They are associated with poor nutritional status and stunted growth in children in LMICs [50, 51]. Different studies have shown associations between parasitic infection and higher Lactulose to Mannitol ratios, indicating increased intestinal permeability [10, 48, 52]. Although we did not perform the Lactulose to Mannitol ratio test, we found a strong correlation between the total number of parasitic infections and an increased fecal level of MPO in children at 24 months of age, further supporting the association between enteric infections and mucosal inflammation. Other studies have also shown similar results. For example, McCormick et al. showed the association of *Giardia* infection with neopterin (NEO), another fecal marker of enteropathy [53]. Both MPO and NEO follow similar concentration trends during the first two years of life in children and are collinear to each other [26, 27, 53]. α-1-antitrypsin (AAT), a fecal biomarker of intestinal inflammation, is positively associated with enteropathogen exposure and diarrhea [53].

We also observed that the fecal levels of MPO correlated with the total number of diarrheal episodes and the duration of diarrheal days. Previous studies have shown that enteric infections in LMICs contribute to malnutrition associated diarrhea [1]. Diarrheal diseases from ETEC, rotavirus, *V*. *cholerae* O1, and *C*. *jejuni* in Bangladeshi children are common, and these enteric infections consequently lead to EE [29, 54].

A previous study showed by measuring EndoCab antibodies in serum that stunting at 12 months of age is associated with prolonged diarrhea and gut barrier dysfunction in Bangladeshi children [1]. A similar study carried out in Gambian infants showed a correlation of EndoCab concentrations with growth faltering [21]. In our longitudinal study of a birth cohort, we found that children who were malnourished (wasted, stunted, or underweight) at 18 months of age had higher EndoCab levels at an earlier stage of life (3 months of age). This result suggests that EndoCab levels early in life, likely from exposure to endotoxin from enteric infections, might predict subsequent malnutrition in children [1].

We also observed that children who were malnourished or wasted at 18 months of age had higher levels of fecal MPO and sCD14 in serum at an earlier stage of life (3 months of age), consistent with prior study findings [24, 53, 55, 56]. Although the impact of sCD14 on nutritional status is not well known, it plays a role in the activation of the endotoxin signaling cascade [9]. Intestinal barrier dysfunction due to constant exposure to endotoxin decreases the absorptive surface area. It increases the permeability of the gut, impairing the digestive and absorptive process and eventually causing wasting and stunting of growth [57]. Our findings provide evidence that EndoCab and sCD14 levels in serum and fecal MPO levels are useful measures of EE and are associated with the subsequent nutritional status of children.

There were several limitations to our study. First, although biopsy studies are underway now in Bangladesh [58], we did not take intestinal biopsy samples from the participants in this birth cohort study, as studying EE was not our primary objective [29]. Second, the sample size was small (43 samples) as we utilized the stored samples of a previous study for our current analyses [29]. We did not have enough availability of samples for all the 43 individuals to evaluate the markers. So, the number of samples varied in different analyses of the markers. Third, we relied on microscopic detection of parasites in the fecal samples in the original study, although recent studies have demonstrated that quantitative real-time PCR (qPCR) is more sensitive [59].

Despite these limitations, we believe that the results suggest that biomarkers of EE like fecal MPO and serum EndoCab and sCD14 levels in children at early ages are predictive of subsequent malnutrition and that MPO, in particular, is a marker of the burden of previous enteric infections. Our study findings strengthen the reliability of using EndoCab, sCD14, and MPO as biomarkers of EE. The use of MPO as a biomarker of EE might help in the early identification of the disease condition indirectly and non-invasively and can predict the overall nutritional status in children before they become malnourished. Future studies with a large sample size will be needed to assess the relationship between biomarkers and immune responses to enteric infections and vaccination in the LMICs like Bangladesh.
